# Catalytically Active Sites on Ni_5_P_4_ for Efficient Hydrogen Evolution Reaction From Atomic Scale Calculation

**DOI:** 10.3389/fchem.2019.00444

**Published:** 2019-06-17

**Authors:** Jun Hu, Xiaofei Cao, Xin Zhao, Wei Chen, Guo-ping Lu, Yong Dan, Zhong Chen

**Affiliations:** ^1^School of Chemical Engineering, Northwest University, Xi'an, China; ^2^School of Materials Science and Engineering, Nanyang Technological University, Singapore, Singapore; ^3^School of Pharmaceutical and Chemical Engineering, Taizhou University, Taizhou, China; ^4^School of Chemical Engineering, Nanjing University of Science and Technology, Nanjing, China

**Keywords:** nickel phosphides, water splitting, hydrogen evolution reaction, density functional theory, machine learning

## Abstract

Ni_5_P_4_ has received considerable attention recently as a potentially viable substitute for Pt as the cathode material for catalytic water splitting. The current investigation focuses on theoretical understandings of the characteristics of active sites toward water splitting using first-principle calculations. The results indicate that the activity of bridge NiNi sites is highly related on the bond number with neighbors. If the total bond number of NiNi is higher than 14, the sites will exhibit excellent HER performance. For the top P sites, the activity is greatly affected by the position of coplanar atoms besides the bond number. Data of bond length with neighbors can be used to predict the activity of P sites as reviewed by machine learning. Partial density of state (PDOS) analysis of different P sites illustrates that the activity of P sites should form the appropriate bond to localize some 3p orbits of the P atoms. Bond number and position of neighbors are two key parameters for the prediction of the HER activity. Based on the current work, most of the low-energy surfaces of Ni_5_P_4_ are active, indicating a good potential of this materials for hydrogen evolution reactions.

**Graphical Abstract:**
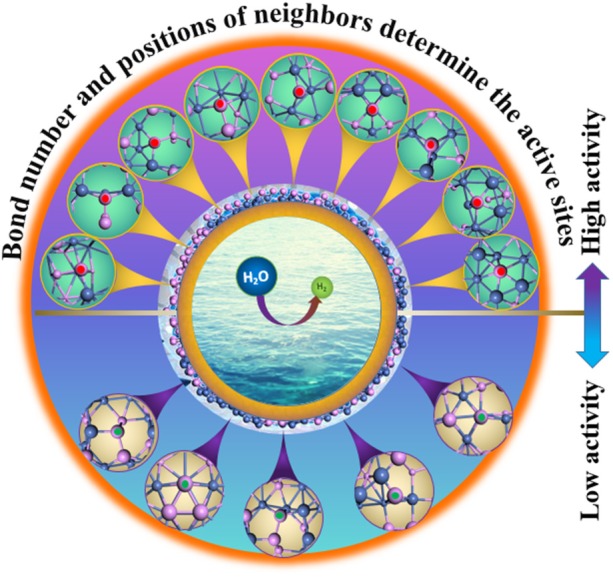
Bond number and relative position with neighbors are two important factors on the activity of Ni_5_P_4_ for HER.

## Introduction

Growing concerns on the energy crisis and environmental problems urgently demand for the development of clean and affordable renewable energy sources as feasible alternatives to the diminishing fossil fuels. Water electrolysis is the most promising option to generate high purity hydrogen as a clean energy source, but unfortunately, the required cathode materials for electrochemical water splitting, such as Pt, is too expensive for large-scale application (Li et al., 2016). This has prompted continued research effort toward searching for earth-abundant elements as the cathode materials for large-scale applications. The potential candidates include transition metals (McKone et al., 2013), and their dichalcogenides (Chen et al., 2011; Voiry et al., 2013; Xie et al., 2013; Yang et al., 2013), carbides (Chen et al., 2013a,b), borides (Vrubel and Hu, 2012), nitrides (Cao et al., 2013), phosphides (Feng et al., 2016), and metal-free carbon nitrides (Merlet et al., 2012; Meng et al., 2017), etc.

Among these materials, nickel phosphides (Ni_*x*_P_*y*_) have shown great promise due to their high activity and stability (Gerasimov and Simirskii, 2008; Laursen et al., 2018). Ni_*x*_P_*y*_ has been reported in more than 10 stoichiometric compositions (Feng et al., 2014; Huang et al., 2014; García-Muelas et al., 2018). Among them, Ni_5_P_4_ has drawn lots of attentions recently on its synthesis, structure, and reactivity (Shu et al., 2005; Zhao et al., 2015). For example, Pan et al. investigated the electrocatalytic property for hydrogen evolution reaction (HER) of Ni_12_P_5_, Ni_2_P, and Ni_5_P_4_, and found that the catalytic property followed the order of Ni_5_P_4_ > Ni_2_P > Ni_12_P_5_ (Pan et al., 2015). Laursen et al. found that the Tafel slope and overpotential at −100 mA cm^−2^ are 33 mV dec^−1^ and −62 mV in 1 M H_2_SO_4_, which are very close to Pt in strong acidic solution (Laursen et al., 2015). Although Ni_5_P_4_ has high activity for HER, an atomic-scale understanding of their reactivity has been elusive, because of the diversity of possible active sites on its different crystal surfaces. While experimental approaches will face a great challenge, theoretical studies can provide insight of the active sites and therefore become an important tool for understanding the catalytic activity of Ni_5_P_4_.

Some researchers believed that the superior performance of Ni_5_P_4_ can be attributed to a high positive charge on Ni atoms and the ensemble effect of P, where the number of Ni3-hollow sites that bind H very strongly is decreased due to the abundance of P, which therefore leads to more thermoneutral adsorption (Liu and Rodriguez, 2005; Liu et al., 2005). However, recent experimental research indicates that NiP_2_ material is also able to exhibit excellent HER activity although there are no Ni3-hollow sites on the surfaces due to the enriched P atoms, as shown in Figure S1 (Jiang et al., 2014; Pu et al., 2017). Recently, Wexler et al. found through simulation that P site was the most active site, but the hollow Ni sites on Ni_2_P and Ni_5_P_4_ (0001) surfaces were not active (Wexler et al., 2017). This result agrees with Jin et al. and our recent work, where we found P sites were suitable for HER for Ni_3_P (Jin et al., 2016; Hu et al., 2018a). However, it was also found not all P sites are active. Therefore, it is necessary to obtain a fundamental understanding of the activity for P sites from the atomic scale, which are important for the development of a broad range of catalytic materials. Till now, no such model exists to reveal an in-depth understanding of the catalytically active sites. Therefore, finding the key parameters affecting the HER activity becomes an essential task for the rational design and optimization of efficient catalysts.

Herein, we report a comprehensive theoretical study on the atomic active sites of Ni_5_P_4_ for HER. It was found that there are three types of active sites, namely the bridge NiNi sites, bridge NiP sites, and top P sites. The activity of these active sites is closely associated with the bond number and position with respect to the neighbors. A direct link between the macroscopic activity and the atomic-scale properties was therefore established by regression and machine learning method based on the generated understandings. The outcome provides not only an improved understanding of Ni_5_P_4_, but also a guideline for the design and synthesis of this material as an electrocatalyst for HER.

## Computational Methods

All calculations were implemented in the CASTEP module of the Materials Studio package (Accelrys Inc., San Diego, CA, USA). During the calculations, self-consistent periodic DFT was adopted by generalized gradient approximation with Perdew-Burke-Ernzerhof exchange-correlation functional. The plane-wave ultrasoft pseudopotential method, describing the ionic cores of Ni-3*d*^8^4*s*^2^ and P-3*s*^3^3*p*^2^, were represented the electron-ion interaction in reciprocal space. The Broyden-Fletcher-Goldfarb-Shanno (BFGS) scheme was selected as the minimization algorithm. The energy cutoff is 380 eV and the SCF tolerance is 5.0 × 10^−7^ eV atom^−1^. The k-points samplings is set as 1 × 1 × 1 for different surfaces. The optimization is completed when the energy, maximum force, maximum stress and maximum displacement are smaller than 5.0 × 10^−6^ eV atom^−1^, 0.01 eV Å^−1^, 0.02 GPa, and 5.0 × 10^−4^ Å, respectively. These parameters were verified by experimental data, as listed in Table S1 and Figure S2, and our previous calculations (Hu et al., 2018a).

The surfaces, containing at least six layers, were obtained from the bulk Ni_5_P_4_ (space group *hP*36, 186, as shown in Figure S3) with a vacuum region of 15 Å. Considering the symmetry of bulk Ni_5_P_4_, seven low-index surfaces, viz., the (001), (100), (110), (1$\bar{1}$0), (101), (111), and (1$\bar{1}$1), with different terminations were chosen during calculation. Different terminations are indicated using capital letters A, B, C, D, and E (more information can be found in Figures S4, S5). It was found that only P-rich and stoichiometric surfaces are stable in all low-index surfaces (Figure S6). Based on the surface energies, seven low energy surfaces (details in Figure S7) are selected for the investigation of the catalytic activity. The energies of species related to the calculation can be found in Table S2.

The Gibbs free energy of adsorption hydrogen atom is calculated by Equation (1) (Hu et al., 2018b):

(1) $$\begin{array}{l} \Delta G_{\text{H}}=E[{\text{Ni}}_{5}{\text{P}}_{4}+\text{H}]-E[{\text{Ni}}_{5}{\text{P}}_{4}]-1/2E[{\text{H}}_{2}]+\Delta E_{\text{ZPE}}\\ \;-T\Delta {\text{S}}_{\text{H}} \end{array}$$

where *E*[Ni_5_P_4_+H] is the total energy of the system, including the adsorbed molecules and the Ni_5_P_4_ facet; *E*[Ni_5_P_4_] is the energy of Ni_5_P_4_ facet; *E*(H_2_) represents the total energy of a gas phase H_2_ molecule; ΔE_ZPE_ denotes the zero-point energy of the system simplified as 0.05 eV. The term –TΔS_H_ is the contribution from entropy at temperature K, taken as 0.20 eV at 298 K (Tang and Jiang, 2016).

## Results and Discussion

### Active Sites for HER

**Figure 1:**
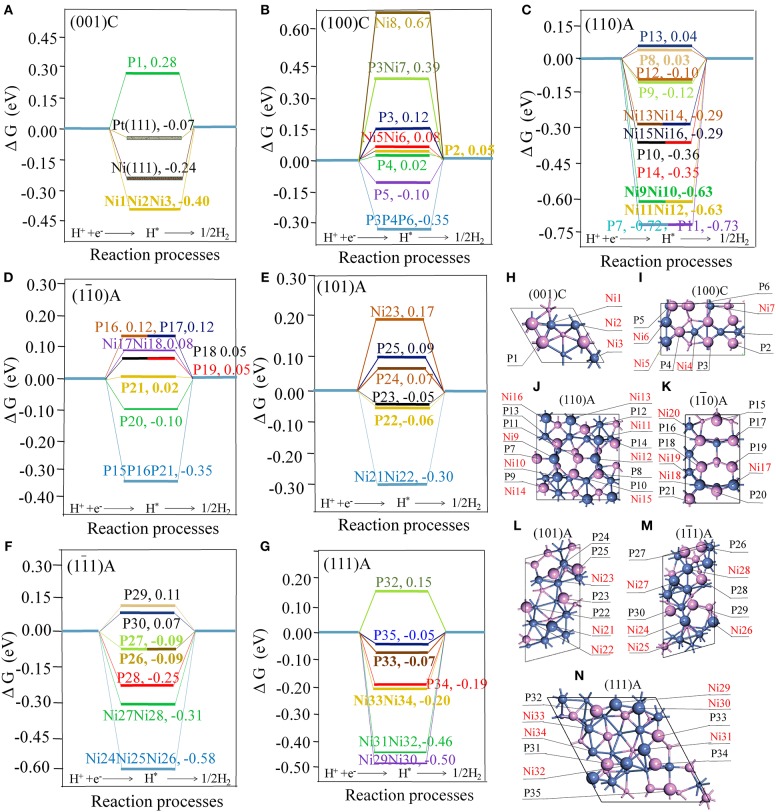
Free energy profile of H_2_ generation on different active site of surfaces with the units in eV **(A)** the (001)C surface, with Pt(111) and Ni(111) surfaces included for comparison; **(B)** the (100)C surface; **(C)** the (110)A surface; **(D)** the (1$\bar{1}$0) A surface; **(E)** the (101)A surface; **(F)** the (1$\bar{1}$1) A surface; **(G)** the (111)A surface. The geometry structures of different surfaces **(H)** the (001)C surface **(I)** the (100)C surface; **(J)** the (110)A surface; **(K)** the (1$\bar{1}$0) A surface; **(L)** the (101)A surface; **(M)** the (1$\bar{1}$1)A surface; **(N)** the (111)A surface; Violet spheres stand for P atoms and blue spheres stand for Ni atoms. Large, medium, and small spheres stand for the atoms located in the first, second, and third layer.

ΔG_H_ is considered as a good descriptor of materials for catalyzing hydrogen generation following either the Volmer-Tafel or the Heyrovsky mechanism (Hinnemann et al., 2005). In principle, smaller of |ΔG_H_| means better HER activity (Zheng et al., 2014). Figure 1 displays details of the adsorption energies involved in the water splitting process on the different sites.

**Figure 2:**
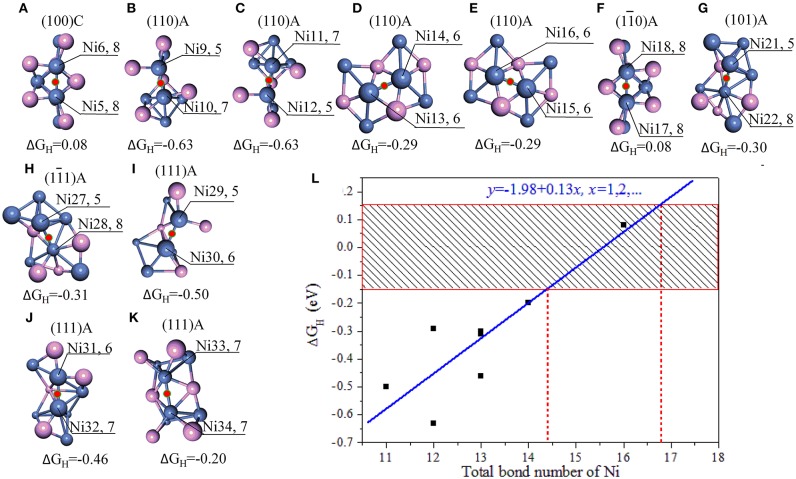
**(A–K)** Stable adsorption sites with bridge NiNi; **(L)** the relationship between the bond number of Ni and ΔG_H._ The bonds are marked when the bond tolerance changes from 0.600 to 1.185, and violet spheres stand for P atoms and blue spheres stand for Ni atoms. In order for easy comparison, the ΔG_H_ are also manifested in the units of eV.

**Figure 3:**
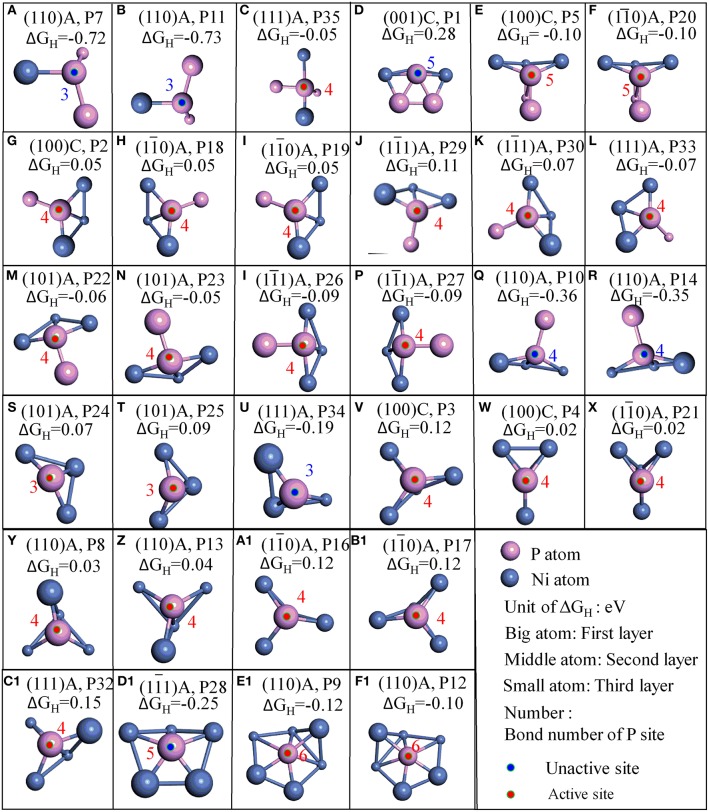
Stable adsorption sites with surface P atoms; **(A–F)** surface P sites with 2 PP bonds; **(G–R)** surface P sites with 1 PP bonds; **(S–F1)** surface P sites with 0 PP bonds; The bond number (red number) of P are marked when the bond tolerance changes from 0.600 to 1.185.

As illustrated in Figure 1H, we found that ΔG_H_ for Ni(111) and Pt(111) surfaces are −0.23 and −0.07 eV, respectively. This is largely consistent with previous experimental and calculated results (Nørskov et al., 2005; Tan et al., 2013). As seen from Figure 1, some hollow NiNiNi sites, hollow PPP sites, bridge NiNi sites, and top P sites are able to stably adsorb the hydrogen atom (H^*^). If we consider a site is active when the |ΔG_H_| is smaller than 0.15 eV, the hollow sites are not catalytically active while some bridge NiNi sites and top P sites may be active. In order to reveal the characteristic of the active sites, detailed structures for bridge NiNi sites and top P sites are illustrated in Figures 2, 3.

As indicated in Figures 2A–K, only a few bridge NiNi sites are active, such as the bridge Ni5Ni6 and Ni17Ni18 while others are not. It is notable the linear correlation coefficient is 0.869 between total bond number of Ni and ΔG_H_, and the linear fit between bond number of Ni (*x*) and ΔG_H_ (*y*) obeys *y* = −1.98 + 0.13*x*. Therefore, the bond number plays an important role to the activity and it may exhibit excellent HER performance when the total bond number of Ni equals to 15 or 16 (Figure 2L).

Furthermore, it is interesting to notice that the ΔG_H_ for most top P sites are closer to zero (P2, P3, P4, P5, P8, P9, P12, P13, P16, P17, P18, P19, P20, P21, P22, P23, P24, P25, P26, P27, P29, P30, P33, and P35). It means these sites are able to trap protons and bond the atomic hydrogen while still able to desorb H_2_ easily. This finding agrees well with the results reported by Jin et al., where it was reported that top P may be the active sites for Ni_3_P (Jin et al., 2016). Additionally, other top P sites are not suitable for HER such as P1, P7, P10, P11, P14, P28, P32, and P34. In order to clearly recognize the key parameters affecting the activity, more detailed geometry structures of P sites are plotted in Figure 3.

As indicated in Figure 3, P sites can be divided into three types based on the number of PP bond during the surface cleavage. The first type is the one that has two PP bonds, these P sites are active if one of the PP bond is a double bond (P35, P5, and P20) while the other is not (P7, P11, and P1). The second type is the P sites with 1 PP bond. All such P sites have three bond number with Ni. These P sites are active if only one neighboring atom (regardless of Ni or P atom) is coplanar with this P site (P2, P18, P19, P29, P30, P33, P22, P23, P26, and P27). While the P sites are not active if the two neighboring atoms are coplanar with the P site. For example, P10 and P14 are coplanar with one neighboring Ni and one neighboring P. The third is the P site without PP bond. This type of P sites have 3, 4, 5, and 6 bond number with Ni. For the P sites with three bond number, the P sites are active if no neighboring atom is coplanar with this P site (P24, P25), while it is not active if one neighboring atom is coplanar with this P site (P34). Furthermore, P sites with four or six bond number are active while P sites with five bond number are not active. The information thus displays a “structure sensitivity” of this material. The determination of the bond number on the activity has also been found in other catalysts (Zhao et al., 2016; Wang et al., 2018). In this work, not only the bond number but also the relative position with neighbors play an important role to determine the activity of P atom for HER (Graphical Abstract).

**Figure 4:**
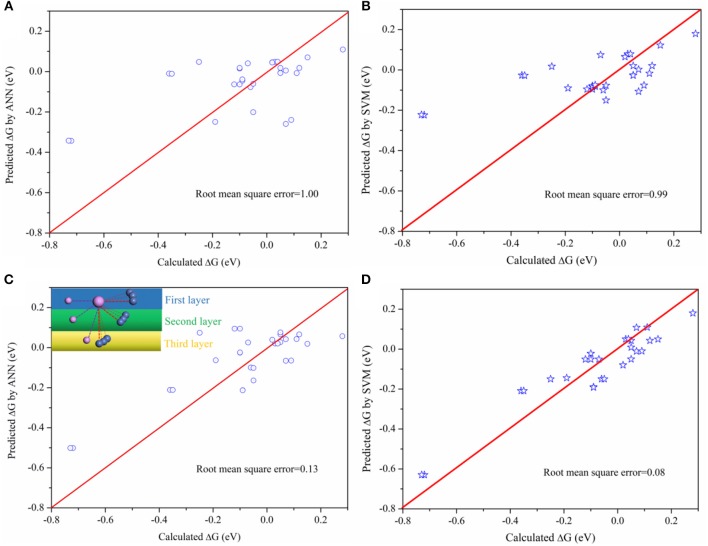
Comparing the predicted ΔG with calculated ΔG on P sites **(A)** the predicted ΔG by ANN method based on the BL data, arranged from largest to smallest; **(B)** the predicted ΔG by SVM method based on the BL data; **(C)** The predicted ΔG by ANN method based on the BLP data; **(D)** the predicted ΔG by SVM method based on the BLP data.

Although this connection between activity and bond property is obvious, it is difficult to fit by mathematical models due to the complexity. Machine learning method, which is capable of analyzing complex data, is used to analyze the results obtained. Two machine learning methods, the Artificial Neural Network (ANN) and Support Vector Machine (SVM), were used to predict the activity. During the ANN training, the number of input layer, middle layer, and output layer were chosen based on the characteristics of the data. This data were randomly split into training (50%) and testing (50%) groups to prevent overtraining. For the SVM model, the regularization parameter is set as 10 in order to balance the classification accuracy and overfitting for the training data. The Kernel type is Radial Basis Function (RBF) with RBF gamma equals to 0.1. Furthermore, the model will stop the optimization when the error between the adjacent steps is <0.1%. The results are shown in Figure 4. There are two database during the training, one for the bond length (BL) (Table S3), where the bond length of active sites are arranged from long to short, and the other the bond length–position (BLP) (Table S4), arranged from the first, second, and third layer based on position of active P site as indicated in the inset image of Figure 4C.

As indicated in Figure 4, there is a larger root mean square error based on BL data while the error will be greatly reduced based on BLP data. The different between BLP and BL data is only whether the data contains the information of relative positions or not. The result verifies our early finding (Figure 3) that the positions of neighboring atoms indeed play an important role on the catalytic activity. Furthermore, The SVM model is more appropriate for the catalytic activity prediction than the ANN model. Therefore, this work demonstrates that it is possible to establish a relationship between activity (macroscopic activity) of P sites and their bond length (an atomic-scale property) by using appropriate machine learning method. This method is potentially useful for high-throughput calculations because it can drastically reduce the amount of calculations.

### Electronic Characteristics of Active Sites

**Figure 5:**
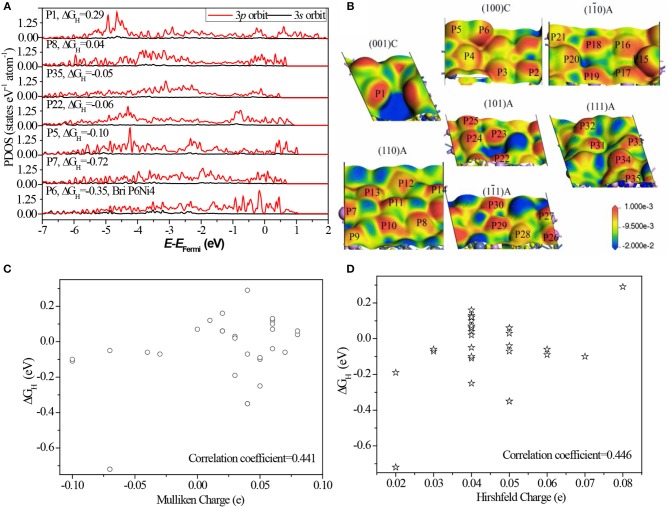
**(A)** The corresponding PDOS of P atoms. The corresponding PDOS of all P sites can be found in Figure S8; **(B)** the electron density difference map of different surfaces with potential isovalue equals to 2.0 eV are inserted in, where a loss of electrons is indicated in blue and electron enrichment is indicated in red; **(C)** the relationship between Mulliken charge and ΔG_H_; **(D)** the relationship between Hirshfeld charge and ΔG_H_; Details data of charge can be found in Table S5.

As indicated above, most of P sites are active sites in Ni_5_P_4_. To reveal the origin of HER activity, partial density of states (PDOS) of different P sites are illustrated in Figure 5.

Comparing the PDOS of different sites (Figure 5A and Figure S8), we found the separated 3*p* orbits, one localized in the region of −5.0 to −3.0 eV and another localized in the region of −1.0 to 1.0 eV, have a high activity. While greatly localized 3*p* orbits near the Fermi level (P6) and deeper energy level (P1) are not active. This finding suggests that the activity of P sites comes from the appropriate bond to localize some 3*p* orbits of the P atom: weakly bonded or strongly bonded P atom weakens its activity. The weakly bonded P sites, such as P6 and P7, tend to form strong stable bonds with H^*^, where the atoms have few electron-state fluctuations and the charge-transfer is more common. Strongly bonded P sites, such as P1, tend to form weak but stable bonds with H^*^ and make it impossible to form H bond on the sites (Falicov and Somorjai, 1985).

Based on the electron density difference map (Figure 5B), the catalytic activity has a strong relation with the electron distribution. The same distribution has almost same activity, for example, between P6 and P15, and P2 and P18. As known, the neighboring bonds largely affect the electron distribution, therefore, the activity of P sites are greatly related to the bond number and relative position of neighbors. Furthermore, the relationship between charge states with ΔG_H_ was also analyzed. Some literatures indicated that the catalytic activity has a strong relation with charge of the surface atom (Balteanu et al., 2004). Statistical analysis (Figures 5C,D) shows that the linear correlation coefficient is only 0.441 between Mulliken charge and ΔG_H_, and 0.446 between Hirshfeld charge and ΔG_H_. This indicates that there is no strong relationship between charge states and HER activity of Ni_5_P_4_. The main reason is the charge states do not contain the relative position with neighbors while charge distribution includes this information.

## Conclusions

In this paper, a comprehensive theoretical analysis is presented on the catalytic characteristics of different active sites of Ni_5_P_4_ for electrochemical water splitting. The results indicate the bond number and relative position with neighbors play an important role on the activity of Ni_5_P_4_ for HER. There are two active sites, namely the bridge NiNi sites and top P sites. The bridge NiNi sites with a total bond number of Ni equals to 15 or 16 exhibit good HER performance. For the top P sites, the activity is greatly affected by bond number of P as well as the coplanar atoms. Data on the bond length with neighbors can be used to predict the activity of P sites as reviewed by machine learning. PDOS of different P sites illustrates that the activity of P sites should form the appropriate bond to localize some 3p orbits of P atom. Weakly bonded or strongly bonded P atom will weaken its activity. Therefore, bond number and positions of neighbors are two key parameters for HER activity of Ni_5_P_4_ material. The current work establishes a clear connection between the macroscopic activity and geometrical structures of Ni_5_P_4_ material. The outcome not only provides important insights into the surface activity for water splitting, but also opens up an exciting opportunity to quickly design and optimize the materials with high catalytic activity. Except the (001)C that is non-active, most of the investigated surfaces of Ni_5_P_4_, e.g., the (100)C, (110)A, (1$\bar{1}$0)A, (101)A, are active for HER, indicating that this material is a good candidate for practical hydrogen production.

## Data Availability

The raw data supporting the conclusions of this manuscript will be made available by the authors, without undue reservation, to any qualified researcher.

## Author Contributions

JH and ZC conceived and designed the calculations. XC, WC, and YD performed the calculations. XZ and GL analyzed the data. JH and ZC revised the paper.

### Conflict of Interest Statement

The authors declare that the research was conducted in the absence of any commercial or financial relationships that could be construed as a potential conflict of interest.
