# Supplementary material for: Catalytically Active Sites on Ni5P4 for Efficient Hydrogen Evolution Reaction From Atomic Scale Calculation
Source: Front Chem. 2019 Jun 17;7:444. doi: 10.3389/fchem.2019.00444 (PMC6590065; doi:10.3389/fchem.2019.00444)
Supplement: Supplementary file 1 [file Data_Sheet_1.docx]

**Supplementary Material**

Catalytically Active Sites on Ni_5_P_4_ for Efficient Hydrogen Evolution Reaction from Atomic Scale Calculation

# Jun Hu^1, 2^, Xiaofei Cao^1^, Xin Zhao^2^*, Wei Chen ^3^, Guo-ping Lu^2, 4^, Yong Dan^1^, Zhong Chen^2^*

^1^ School of Chemical Engineering, Northwest University, Xi’an, China

^2^ School of Materials Science and Engineering, Nanyang Technological University, Singapore, Singapore

^3^ School of Pharmaceutical and Chemical Engineering, Taizhou University, Taizhou, China

^4^ School of Chemical Engineering, Nanjing University of Science & Technology, Nanjing, China

**ORCID**

J. Hu: 0000-0002-3075-9291

X. Zhao: [0000-0002-7493-1014](http://orcid.org/0000-0002-7493-1014)

W. Chen: 0000-0002-1233-9023

Z. Chen: 0000-0001-7518-1414

**Figure S1.** The crystallographic structure of NiP_2_ (space group *C2/c*, 15), A and B indicate different terminations. There are no Ni3-hollow sites on the surfaces due to the enriched P atoms.

**Figure S2.** Free energy profile of H_2_ generation on the (110)A, P8 active site of surfaces. It indicates there are few differences for the free energy of adsorption H* when the cutoff energy is 380 eV and K-point is 1×1×1.


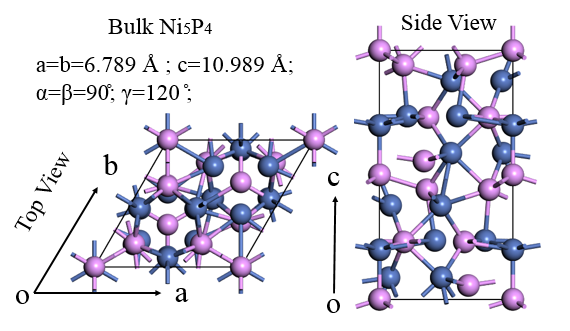


**Figure S3.** The crystallographic structure of Ni_5_P_4_ (space group *hP*36, 186).


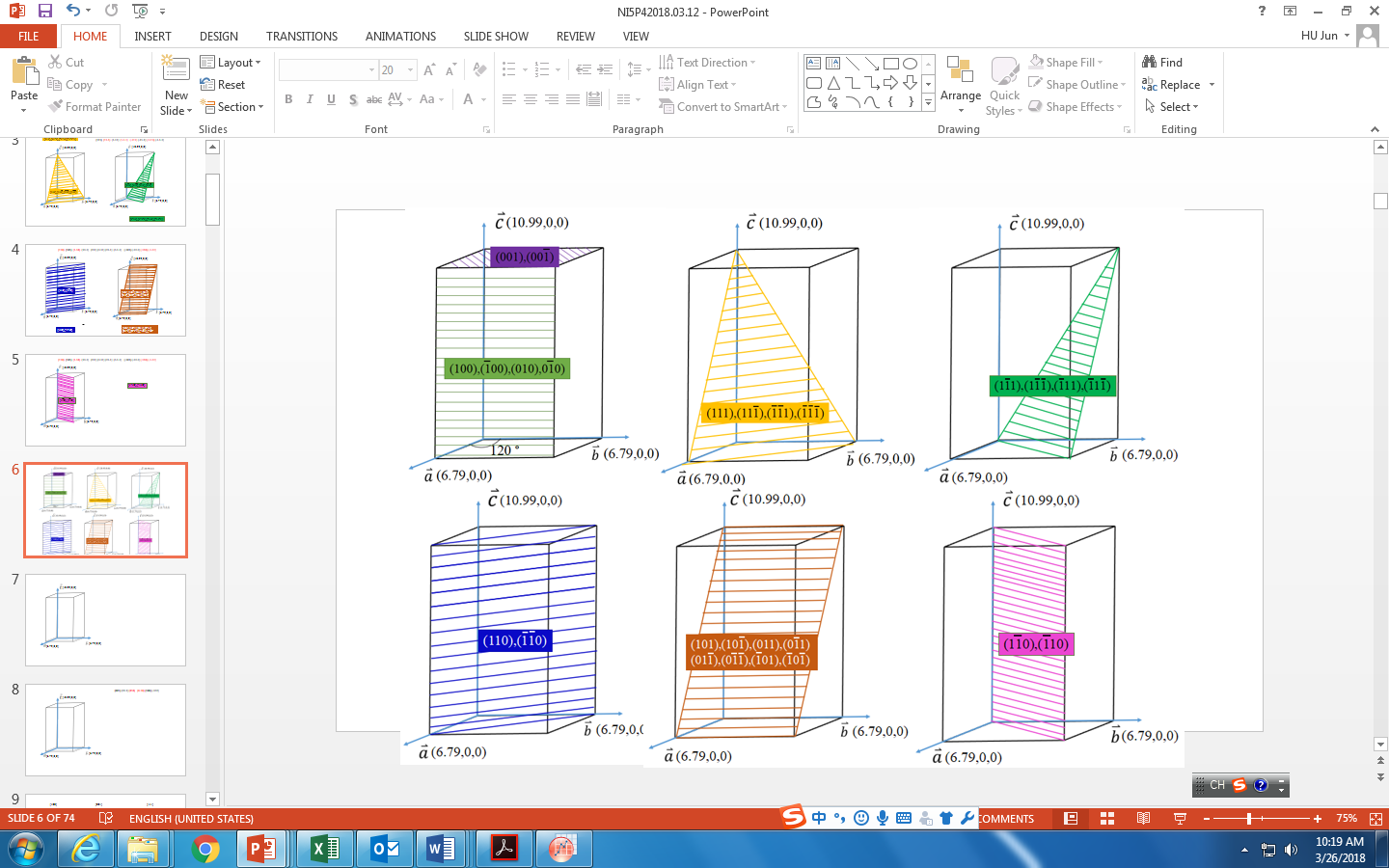


**Figure S4.** All surfaces considered in this paper, where equivalent surfaces (family of planes) are put together. Based on the symmetry of Ni_5_P_4_, we selected the (001), (100), (111), (1$\bar{1}$1), (110), (101) and (1$\bar{1}$0) surfaces for the calculation.


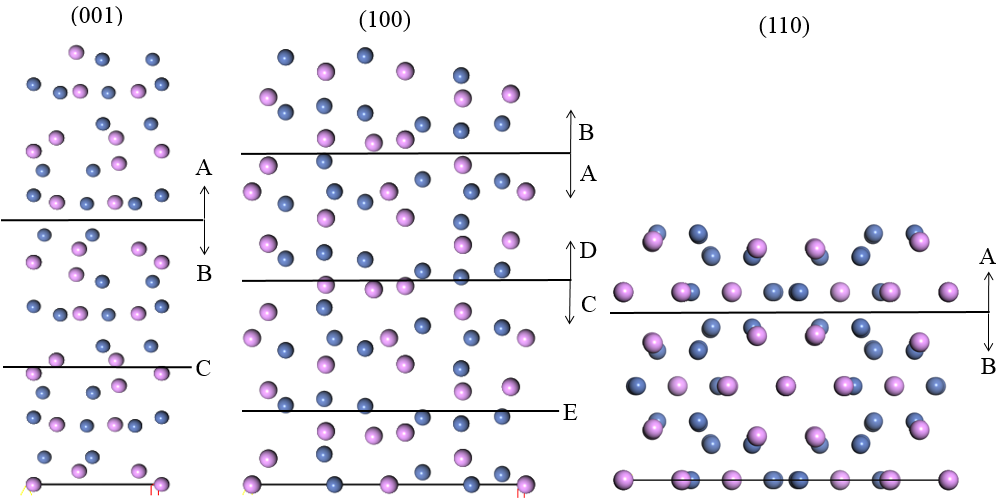


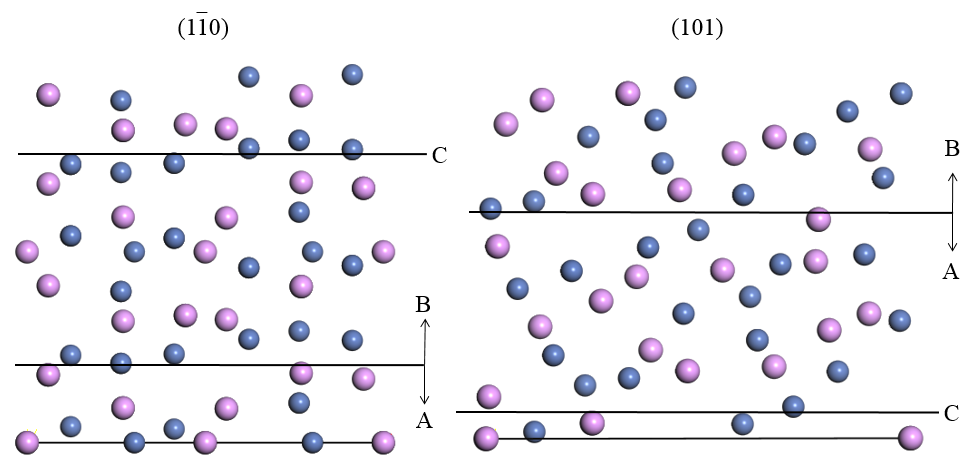


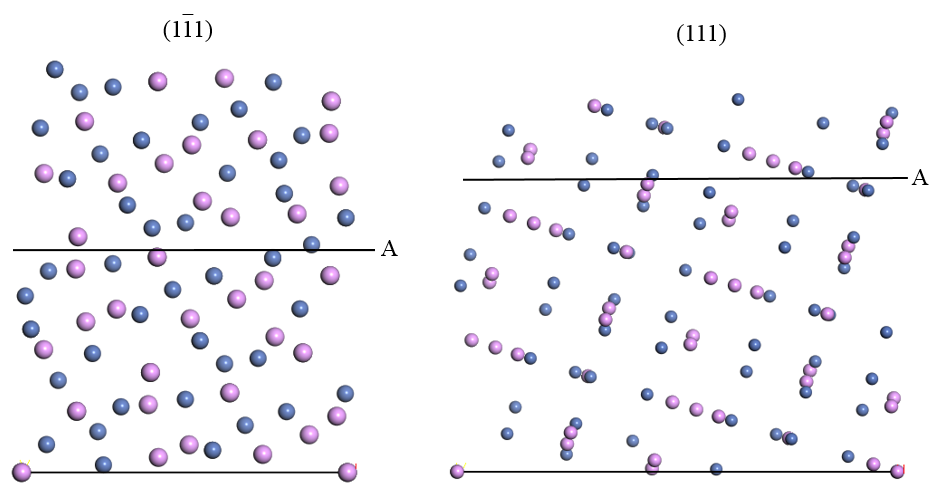


**Figure S5.** The terminated position on different surfaces.

**Figure S6.** The energy of surfaces with different terminations. (a) the (001) facet; (b) the (100) facet; (c) the (110) facet; (d) the (10) facet; (e) the (101) facet; (f) the (111) and (11) facets. The surface energy can be calculated by equation: $E_{sur}=\frac{1}{2A}(E_{slab}-n_{Ni}E_{bulk Ni}-n_{P}E_{bulk P}+\frac{E_{form}}{5}n_{Ni}+{\Delta\mu}_{P}(\frac{{4n}_{Ni}}{5}-n_{P}))$.^[^[[1]](#endnote-1)^]^ Where *E*_slab_ is the total energy of the slab model, *E*_bulk Ni_ and *E*_bulk P_ are the total energy per atom of the metal Ni *fcc* and black P, *E*_form_ is the formation energy of Ni_5_P_4_, $n_{Ni}$ and $n_{P}$ are the number of atoms in the slab model, and A is the surface area of the slab model. Furthermore, the chemical potentials of P (Δ*μ_P_*) is determined by the equilibrium equation: Δ*G_NixPy_ = x*Δ*μ_Ni_ + y*Δ*μ_P_* at 0 K based on different nickel phosphide phases. The results show that only P-rich and stoichiometric surfaces have low surface energies among all low-index surfaces. The lowest terminations on different surfaces were selected to investigate the activity as they are the most stable surfaces.

**Figure S7.** The geometric structure of seven stable surfaces. The atoms on the first layer of surface are shown in big spheres, second layer of surface in medium-sized spheres, the third layer of surface in small spheres and others are shown in sticks. Violet spheres stand for P atoms and blue spheres stand for Ni atoms.

**Figure S8**. The corresponding partial density of states (PDOS) of different P sites (a) P1 to P6; (b) P7 to P12; (c) P13 to P18; (d) P19 to P24; (e) P25 to P30; (f) P31 to P35.

**Table S1.** Optimized crystallographic parameters of Ni_5_P_4_ compared to experimental data. As shown in Table S1, the simulated crystallographic parameters of the optimized Ni_5_P_4_ agree well with the experimental results from Powder X-ray diffraction.

|  | Crystallographic parameters | | | |
| --- | --- | --- | --- | --- |
|  | *A*(Å) | *b*(Å) | *c*(Å) | *γ*/(°) |
| This work | 6.789 | 6.789 | 10.989 | 120 |
| Powder X-ray diffraction Cu Kα ^S^[[2]](#endnote-2)^^ | 6.78944 | 6.78944 | 10.9891 | 120 |
| Powder X-ray diffraction Cu Kα ^S^^[[3]](#endnote-3)^ | 6.8011 | 6.8011 | 11.0064 | 120 |
| Powder X-ray diffraction Cu Kα ^S^[[4]](#endnote-4)^^ | 6.760 | 6.760 | 10.988 | 120 |

**Table S2.** The calculated energies of different species.

| Species | Energy / eV.atom^-1^ | Species | Formation Energy / eV.atom^-1^ |
| --- | --- | --- | --- |
| Bulk Ni | -1355.276 | Ni_3_P | -0.445 |
| Black P | -179.674 | Ni_8_P_3_ | -0.461 |
| Bulk Ni_5_P_4_ | -7500.101 | Ni_12_P_5_ | -0.481 |
| H_2_ | -31.548 | Ni_2_P | -0.478 |
|  |  | Ni_5_P_4_ | -0.481 |
|  |  | NiP | -0.436 |
|  |  | NiP_2_ | -0.376 |
|  |  | NiP_3_ | -0.304 |
|  |  | NiP_4_ | -0.168 |

**Table S3.** The bond length (BL) data of different P sites for Artificial Neural Network and Support Vector Machine.

| Sites | PP bond1 | PP bond2 | PNi bond1 | PNi bond2 | PNi bond3 | PNi bond4 | PNi bond5 | PNi bond6 |
| --- | --- | --- | --- | --- | --- | --- | --- | --- |
| P1 | 2.333 | 2.325 | 2.279 | 2.273 | 2.420 | 0.000 | 0.000 | 0.000 |
| P2 | 2.203 | 0.000 | 2.322 | 2.314 | 2.256 | 0.000 | 0.000 | 0.000 |
| P3 | 0.000 | 0.000 | 2.317 | 2.264 | 2.248 | 2.22 | 0.000 | 0.000 |
| P4 | 0.000 | 0.000 | 2.268 | 2.261 | 2.205 | 2.205 | 0.000 | 0.000 |
| P5 | 2.557 | 2.236 | 2.415 | 2.415 | 2.130 | 0.000 | 0.000 | 0.000 |
| P7 | 2.227 | 2.195 | 2.300 | 0.000 | 0.000 | 0.000 | 0.000 | 0.000 |
| P8 | 0.000 | 0.000 | 2.269 | 2.245 | 2.237 | 2.179 | 0.000 | 0.000 |
| P9 | 0.000 | 0.000 | 2.513 | 2.371 | 2.36 | 2.298 | 2.241 | 2.218 |
| P10 | 2.196 | 0.000 | 2.338 | 2.234 | 2.29 | 0.000 | 0.000 | 0.000 |
| P11 | 2.227 | 2.196 | 2.299 | 0.000 | 0.000 | 0.000 | 0.000 | 0.000 |
| P12 | 0.000 | 0.000 | 2.514 | 2.370 | 2.359 | 2.298 | 2.241 | 2.219 |
| P13 | 0.000 | 0.000 | 2.269 | 2.245 | 2.236 | 2.179 | 0.000 | 0.000 |
| P14 | 2.195 | 0.000 | 2.339 | 2.29 | 2.233 | 0.000 | 0.000 | 0.000 |
| P16 | 0.000 | 0.000 | 2.317 | 2.264 | 2.247 | 2.221 | 0.000 | 0.000 |
| P17 | 0.000 | 0.000 | 2.317 | 2.264 | 2.247 | 2.221 | 0.000 | 0.000 |
| P18 | 2.203 | 0.000 | 2.323 | 2.314 | 2.256 | 0.000 | 0.000 | 0.000 |
| P19 | 2.203 | 0.000 | 2.323 | 2.314 | 2.256 | 0.000 | 0.000 | 0.000 |
| P20 | 2.572 | 2.234 | 2.414 | 2.414 | 2.133 | 0.000 | 0.000 | 0.000 |
| P21 | 0.000 | 0.000 | 2.27 | 2.261 | 2.207 | 2.207 | 0.000 | 0.000 |
| P22 | 2.212 | 0.000 | 2.423 | 2.295 | 2.263 | 0.000 | 0.000 | 0.000 |
| P23 | 2.212 | 0.000 | 2.415 | 2.274 | 2.230 | 0.000 | 0.000 | 0.000 |
| P24 | 0.000 | 0.000 | 2.224 | 2.2 | 2.133 | 0.000 | 0.000 | 0.000 |
| P25 | 0.000 | 0.000 | 2.188 | 2.212 | 2.157 | 0.000 | 0.000 | 0.000 |
| P26 | 2.219 | 0.000 | 2.371 | 2.318 | 2.191 | 0.000 | 0.000 | 0.000 |
| P27 | 2.219 | 0.000 | 2.363 | 2.318 | 2.199 | 0.000 | 0.000 | 0.000 |
| P28 | 0.000 | 0.000 | 2.35 | 2.315 | 2.225 | 2.21 | 2.167 | 0.000 |
| P29 | 2.217 | 0.000 | 2.348 | 2.276 | 2.268 | 0.000 | 0.000 | 0.000 |
| P30 | 2.209 | 0.000 | 2.333 | 2.267 | 2.261 | 0.000 | 0.000 | 0.000 |
| P32 | 0.000 | 0.000 | 2.241 | 2.231 | 2.213 | 2.212 | 0.000 | 0.000 |
| P33 | 2.248 | 0.000 | 2.341 | 2.156 | 2.136 | 0.000 | 0.000 | 0.000 |
| P34 | 0.000 | 0.000 | 2.222 | 2.185 | 2.135 | 0.000 | 0.000 | 0.000 |
| P35 | 2.24 | 2.187 | 2.585 | 2.219 | 0.000 | 0.000 | 0.000 | 0.000 |

**Table S4.** The bond length–position (BLP) data of different P sites for Artificial Neural Network and Support Vector Machine.

| Sites | PP bond  First layer | PP bond  First layer | PP bond  Secondlayer | PP bond  Third layer | PNi bond  First layer | PNi bond  First layer | PNi bond  First layer | PNi bond  Second layer | PNi bond  Second layer | PNi bond  Second layer | PNi bond  Third layer |
| --- | --- | --- | --- | --- | --- | --- | --- | --- | --- | --- | --- |
| P1 | 2.333 | 2.325 | 0.000 | 0.000 | 2.273 | 2.279 | 0.000 | 0.000 | 0.000 | 0.000 | 2.420 |
| P2 | 0.000 | 0.000 | 2.203 | 0.000 | 2.256 | 0.000 | 0.000 | 2.322 | 2.314 | 0.000 | 0.000 |
| P3 | 0.000 | 0.000 | 0.000 | 0.000 | 0.00 | 0.000 | 0.000 | 2.317 | 2.264 | 2.248 | 2.22 |
| P4 | 0.000 | 0.000 | 0.000 | 0.000 | 2.205 | 2.205 | 0.000 | 2.268 | 0.000 | 0.000 | 2.261 |
| P5 | 2.236 | 0.000 | 2.557 | 0.000 | 2.415 | 2.415 | 0.000 | 0.000 | 0.000 | 0.000 | 2.130 |
| P7 | 2.195 | 0.000 | 0.000 | 2.227 | 2.300 | 0.000 | 0.000 | 0.000 | 0.000 | 0.000 | 0.000 |
| P8 | 0.000 | 0.000 | 0.000 | 0.000 | 2.179 | 0.000 | 0.000 | 2.269 | 2.245 | 0.000 | 2.237 |
| P9 | 0.000 | 0.000 | 0.000 | 0.000 | 2.371 | 2.241 | 2.218 | 2.36 | 2.513 | 0.000 | 2.298 |
| P10 | 2.196 | 0.000 | 0.000 | 0.000 | 2.234 | 0.000 | 0.000 | 2.338 | 0.000 | 0.000 | 2.29 |
| P11 | 2.196 | 0.000 | 0.000 | 2.227 | 2.299 | 0.000 | 0.000 | 0.000 | 0.000 | 0.000 | 0.000 |
| P12 | 0.000 | 0.000 | 0.000 | 0.000 | 2.370 | 2.241 | 2.219 | 2.359 | 2.514 | 0.000 | 2.298 |
| P13 | 0.000 | 0.000 | 0.000 | 0.000 | 2.179 | 0.000 | 0.000 | 2.269 | 2.245 | 0.000 | 2.236 |
| P14 | 2.195 | 0.000 | 0.000 | 0.000 | 2.233 | 0.000 | 0.000 | 2.339 | 0.000 | 0.000 | 2.29 |
| P16 | 0.000 | 0.000 | 0.000 | 0.000 | 0.000 | 0.000 | 0.000 | 2.317 | 2.264 | 2.247 | 2.221 |
| P17 | 0.000 | 0.000 | 0.00 | 0.000 | 0.000 | 0.000 | 0.000 | 2.317 | 2.264 | 2.247 | 2.221 |
| P18 | 0.000 | 2.203 | 0.000 | 0.000 | 2.256 | 0.000 | 0.000 | 2.323 | 2.314 | 0.000 | 0.000 |
| P19 | 0.000 | 2.203 | 0.000 | 0.000 | 2.256 | 0.00 | 0.00 | 2.323 | 2.314 | 0.000 | 0.000 |
| P20 | 2.234 | 0.00 | 2.572 | 0.000 | 2.414 | 2.414 | 0.000 | 0.000 | 0.000 | 0.000 | 2.133 |
| P21 | 0.000 | 0.000 | 0.000 | 0.000 | 2.207 | 2.207 | 0.000 | 2.27 | 0.000 | 0.000 | 2.261 |
| P22 | 2.212 | 0.000 | 0.000 | 0.000 | 0.000 | 0.000 | 0.000 | 2.295 | 2.263 | 0.000 | 2.423 |
| P23 | 2.212 | 0.000 | 0.000 | 0.000 | 0.000 | 0.000 | 0.000 | 2.274 | 2.230 | 0.000 | 2.415 |
| P24 | 0.000 | 0.000 | 0.000 | 0.000 | 2.224 | 2.2 | 2.133 | 0.000 | 0.000 | 0.000 | 0.000 |
| P25 | 0.000 | 0.000 | 0.000 | 0.000 | 2.188 | 2.212 | 2.157 | 0.000 | 0.000 | 0.000 | 0.000 |
| P26 | 2.219 | 0.000 | 0.000 | 0.000 | 2.318 | 0.000 | 0.000 | 2.191 | 0.000 | 0.000 | 2.371 |
| P27 | 2.219 | 0.000 | 0.000 | 0.000 | 2.318 | 0.000 | 0.000 | 2.199 | 0.000 | 0.000 | 2.363 |
| P28 | 0.000 | 0.000 | 0.000 | 0.000 | 2.225 | 2.21 | 0.000 | 2.35 | 2.315 | 0.000 | 2.167 |
| P29 | 0.000 | 0.000 | 2.217 | 0.000 | 2.276 | 0.000 | 0.000 | 2.268 | 0.000 | 0.000 | 2.348 |
| P30 | 0.000 | 0.000 | 2.209 | 0.000 | 2.261 | 0.000 | 0.000 | 2.267 | 0.000 | 0.000 | 2.333 |
| P32 | 0.000 | 0.000 | 0.000 | 0.000 | 2.212 | 0.000 | 0.000 | 2.241 | 2.231 | 0.000 | 2.213 |
| P33 | 0.000 | 0.000 | 2.248 | 0.000 | 2.136 | 0.000 | 0.000 | 2.341 | 2.156 | 0.000 | 0.000 |
| P34 | 0.000 | 0.000 | 0.000 | 0.000 | 2.135 | 0.000 | 0.000 | 2.222 | 2.185 | 0.000 | 0.000 |
| P35 | 0.000 | 0.000 | 2.187 | 2.24 | 0.000 | 0.000 | 0.000 | 2.585 | 2.219 | 0.000 | 0.000 |

**Table S5.** The Mulliken and Hirshfeld charge of different P sites.

| Atom | Mulliken Charge  (e) | Hirshfeld Cahrge  (e) | Atom | Mulliken Charge  (e) | Hirshfeld Cahrge  (e) |
| --- | --- | --- | --- | --- | --- |
| P1 | 0.04 | 0.08 | P19 | 0.02 | 0.05 |
| P2 | 0.02 | 0.05 | P20 | 0.05 | 0.06 |
| P3 | 0.06 | 0.04 | P21 | 0.03 | 0.04 |
| P4 | 0.03 | 0.05 | P22 | 0.07 | 0.06 |
| P5 | 0.05 | 0.07 | P23 | 0.06 | 0.05 |
| P6 | -0.03 | 0.03 | P24 | 0.06 | 0.04 |
| P7 | -0.07 | 0.02 | P25 | 0.06 | 0.04 |
| P8 | 0.08 | 0.04 | P26 | 0.05 | 0.04 |
| P9 | -0.10 | 0.04 | P27 | 0.04 | 0.05 |
| P10 | 0.04 | 0.05 | P28 | -0.03 | 0.03 |
| P11 | -0.07 | 0.02 | P29 | 0.01 | 0.04 |
| P12 | -0.10 | 0.04 | P30 | 0.00 | 0.04 |
| P13 | 0.08 | 0.04 | P31 | -0.03 | 0.04 |
| P14 | 0.04 | 0.05 | P32 | 0.02 | 0.04 |
| P15 | -0.03 | 0.03 | P33 | -0.04 | 0.03 |
| P16 | 0.06 | 0.04 | P34 | 0.03 | 0.02 |
| P17 | 0.06 | 0.04 | P35 | -0.07 | 0.04 |
| P18 | 0.02 | 0.05 |  |  |  |

**References**

1. [] J. Hu, S. Zheng, X. Zhao, X. Yao, Z. Chen, *J. Mater. Chem. A* **2018**, doi: 10.1039/C8TA00437D [↑](#endnote-ref-1)
2. [S] Andersson, S. M.: An X-ray Diffraction Study of the Solid Phase Equilibria in Some M-In-P Systems (M= Ni, Pd, Pt). *J. Alloys Compd.*  **1993**, 194, 67-71. [↑](#endnote-ref-2)
3. [S] Schmetterer, C.; Vizdal, J.; Kroupa, A.; Kodentsov, A.; Ipser, H. The Ni-rich Part of the Ni-P-Sn System: Isothermal Sections. *J. Electron. Mater.* **2009**, 38, 2275-2300 [↑](#endnote-ref-3)
4. [S] Yupko, L.M.; Svirid, A.A.; Muchnik, S.V. Phase Equilibria in Nickel-phosphorus and Nickel-phosphorus-Carbon Systems. Soviet Powder Metallurgy and Metal Ceramics (translated from Poroshkovaya Metallurgiya (Kiev)) **1986**, 25, 768-773. [↑](#endnote-ref-4)
